# Analysis of Time-Dependent Brain Network on Active and MI Tasks for Chronic Stroke Patients

**DOI:** 10.1371/journal.pone.0139441

**Published:** 2015-12-14

**Authors:** Da-Hye Kim, Leahyun Kim, Wanjoo Park, Won Hyuk Chang, Yun-Hee Kim, Seong-Whan Lee, Gyu Hyun Kwon

**Affiliations:** 1 Center for Bionics, Korea Institute of Science and Technology, Seoul, Korea; 2 Graduate School of Technology & Innovation Management, Hanyang University, Seoul, Korea; 3 Department of Brain and Cognitive Engineering, Korea University, Seoul, Korea; 4 Department of HCI & Robotics, University of Science and Technology, Seoul, Korea; 5 Department of Physical and Rehabilitation Medicine, Center for Prevention and Rehabilitation, Heart Vascular and Stroke Institute, Samsung Medical Center, Sungkyunkwan University School of Medicine, Seoul, Korea; University of Michigan, UNITED STATES

## Abstract

Several researchers have analyzed brain activities by investigating brain networks. However, there is a lack of the research on the temporal characteristics of the brain network during a stroke by EEG and the comparative studies between motor execution and imagery, which became known to have similar motor functions and pathways. In this study, we proposed the possibility of temporal characteristics on the brain networks of a stroke. We analyzed the temporal properties of the brain networks for nine chronic stroke patients by the active and motor imagery tasks by EEG. High beta band has a specific role in the brain network during motor tasks. In the high beta band, for the active task, there were significant characteristics of centrality and small-worldness on bilateral primary motor cortices at the initial motor execution. The degree centrality significantly increased on the contralateral primary motor cortex, and local efficiency increased on the ipsilateral primary motor cortex. These results indicate that the ipsilateral primary motor cortex constructed a powerful subnetwork by influencing the linked channels as compensatory effect, although the contralateral primary motor cortex organized an inefficient network by using the connected channels due to lesions. For the MI task, degree centrality and local efficiency significantly decreased on the somatosensory area at the initial motor imagery. Then, there were significant correlations between the properties of brain networks and motor function on the contralateral primary motor cortex and somatosensory area for each motor execution/imagery task. Our results represented that the active and MI tasks have different mechanisms of motor acts. Based on these results, we indicated the possibility of customized rehabilitation according to different motor tasks. We expect these results to help in the construction of the customized rehabilitation system depending on motor tasks by understanding temporal functional characteristics on brain network for a stroke.

## Introduction

Strokes, which occur when the vessels are ruptured or blocked, are the second leading cause of death and a major cause of adult disability [1]. Neuro-imaging technology in the form of functional Magnetic Resonance Imaging (fMRI), Magnetoencephalography (MEG), and Electroencephalography (EEG) have been used to identify changes in the brain after a stroke [2–4]. Even though fMRI and MEG are useful to identify the mechanism of a stroke by the high spatial resolution, they have shortcomings, such as low temporal resolution, and require bulky equipment, such as huge devices and shield rooms. Compared to fMRI and MEG, EEG makes it easy to find temporal brain activity due to its high temporal resolution. This characteristics of EEG is useful to analyze the temporal change of the brain activity, which reflects the brain function [5, 6]. Previous studies have analyzed the spectral power of EEG data during motor tasks for the stroke [7, 8]. However, they have focused less on communication among the brain that change organically [9]. The nervous system is a complex network that is able to interact and produce real-time information from multiple external and internal sources. Functional connectivity indicates the statistical functional associations among brain regions in this nervous system [10]. Brain networks change rapidly by reflecting subsets and pathway in various brain regions according to cognitive and behavioral tasks [11, 12]. For these reasons, we focused on the brain network related to functional connectivity of a stroke using EEG.

Next, we hypothesized the brain activity of chronic stroke patients who are in nearly the last stage of rehabilitation, from a different viewpoint compared to previous studies [13–15]. The spontaneous recovery after a stroke occurs within a month after onset [16–19]. Based on these results, we assumed that the chronic state of the stroke is minimally influenced by the outside because the chronic state has lower regeneration of rehabilitation. Based on this assumption, to focus on the motor activity, we found the temporal characteristics of the damaged network after a stroke. For chronic stroke patients, several studies have researched brain networks during each motor execution or motor imagery (MI) task. Fallani et al. observed the brain network properties of the stroke during their finger tapping. Later, Fallani et al. and Yan et al. just analyzed the brain network properties of the MI tasks for their hand movement [20–22]. However, there is a lack of research on two motor tasks together.

The active task, namely motor execution, involves motor intention and physical movement. The MI task refers to the concept of rehearsal for motor function [23]. The activated brain area of the MI task is similar to the area activated by motor execution. The MI task, also, is known as an efficient way to improve motor functions like the active task [24, 25]. However, it is unclear whether two tasks share the same or analogous cognate routes, although the MI task has a similar function with that of the active task. Therefore, we need to find the brain network properties of two motor tasks together and to identify whether the brain network properties of the two motor tasks relate to motor function in the similar area. Next, there was a lack of studies to find the time-dependent characteristics of the brain network because previous studies analyzed a fixed time window as a resting state or repeated movement on the brain network [12, 20, 26–29].

In this study, we focused on the time-dependent characteristics of network properties for a grasping movement in the active and MI tasks. Our main hypotheses were as follows.

First, we hypothesized that the active and MI tasks would have the different brain network properties according to intuitive movement and that the active task would show stronger characteristics of the brain network on motor area than that of the MI task. Previous studies have shown meaningful brain activity in the primary motor cortex during motor tasks [2, 12]. Because our experimental tasks involve motor tasks of the affected hand for stroke patients, we anticipated that the centrality and small-worldness of the brain network would have stronger properties centered on the motor cortex in the active task than in the MI task.

Second, we hypothesized that there are time-dependent changes of the brain network properties. Previous studies have only analyzed networks having a fixed time window during the resting state or repeated movements [12, 20, 26–29]. They did not consider the temporal characteristics of the brain network. Therefore, we need to investigate the time-dependent changes of brain networks during the single movement of grasping. Based on this single performance, we expect to find the time-dependent properties of the brain networks to identify the brain function similarly to EEG spectral power.

Third, we hypothesized that the network properties for a stroke in two motor tasks would correlate with their motor function. In particular, the primary motor cortex of the affected hemisphere in the active task would have stronger correlation with motor function than in the MI task [2].

## Methods

### Participants

Nine chronic stroke patients who had a motor disturbance by unilateral stroke participated in this study (mean age of 53.5 (4.3) years; 6 males; affected upper limb score of Fugl-Meyer assessment (FMA-UL: 46.7)). The inclusion criteria are patients who had had their first ischemic or cerebral hemorrhagic stroke, which lasted over 3 months after onset, and who was between 18 and 70 years old. The exclusion criteria were patients who had an intracranial metal insertion, claustrophobia, pacemakers, or were prohibited from taking MRI. In this study, we got the approval of the Institutional Review Board (IRB) of the Korea Institute of Science and Technology (KIST) and Samsung Medical Center (SMC) (KIST IRB; KIST 2013–009, SMC IRB; SMC 2013-02-091). All participants were asked to carefully read the informed consent form, and they all voluntarily participated in the experiment. We obtained written consent from all participants and analyzed all research materials under IRB guidance.

### Experimental design

We instructed participants to grasp the haptic device using their affected hand in each motor task. A haptic device in this study was controlled by a DSP processor and was connected with stimulus software made by Flash^TM^. The stimulus software was connected to an EEG system (64 ch, sampling rate: 2048 Hz; Active-two, Biosemi^TM^, Amsterdam, Netherlands) and induced synchronization between the haptic device and EEG system. This system was described in detail in a previous study [8].

**Fig 1:**
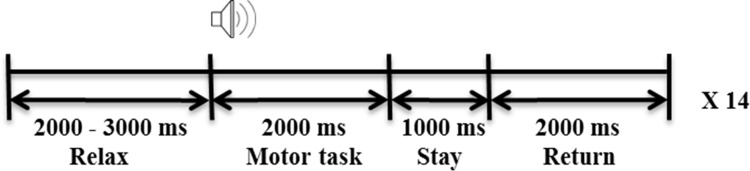
Experimental protocol. An experimental protocol was followed in each trial. Three motor tasks were represented by counter balancing (P-A-M-A-M-P-M-P-A), and each motor task was run three times. Each motor task consisted of 42 trials, and 1 trial took approximately 7 or 8 seconds.

We designed three motor tasks to find the characteristics of each motor task for chronic stroke patients: an active task in which participants move their affected hand by themselves, a passive task in which they move their affected hand by a haptic device, and an MI task that has no movement and the participant just imagines this movement. This experiment involves a total of 42 trials per each motor task. There is a temporal construction of the experiment for each trial (Fig 1).

The haptic device was fixed onto the affected hand of participants by a wrist strap. The other hand remained motionless on the arm of the chair on which the participants were seated. Participants fixed their eyes on the monitor for 2 to 3 seconds and performed each motor task during 2 seconds after a cue. After that, the participants maintained that motor state for 1 second and the haptic device returned to its initial state during 2 seconds.

### EEG data analysis

#### EEG data preprocessing

EEG data were down sampled to 256 Hz and were band-pass filtered between 1 and 80 Hz. After the data of each trial were extracted between -4 and 6 seconds, we removed electrooculography (EOG) and electromyography (EMG) artifacts by the Independent Component Analysis (ICA) using the EEGlab toolbox (Delorme & Makeig, 2004). Next, we removed trials with noise signals caused by the movement or sneezing of participants. Finally, EEG signals were applied to the Common Average Reference (CAR). To find the optimal frequency band related to motor tasks in a brain network, we divided frequency bands that relate to sensorimotor rhythm as mu (8-12Hz), low beta (13-20Hz), high beta (21-30Hz), and gamma (31-50Hz) bands.

#### Brain connectivity

We calculated phase locking value (PLV) of the EEG to find the functional connectivity in the brain network [30]. In a previous study, Sakkalis et al. reviewed the methodology for brain connectivity estimation [31]. They argued that PLV indicates the characteristics of nonlinear, data-driven, stationarity-independent, and functionally-connective. Generally, EEG distribution considers multivariate Gaussian process. This assumption is easily violated during mental and physical activities because the brain state changes with arousal. Therefore, EEG signals have quasi-stationarity. If stationarity is violated, many researchers use PLV, which is a stationarity-independent measurement. Since many crucial neural processes have different nonlinear characteristics, PLV is useful to measure the dynamics of EEG signals. It is also useful to access data-driven systems because the brain is harder to analysis by a predictable model. Therefore, we estimated brain connectivity by PLV, which is useful to analyze the functional connectivity of EEG.

PLV measures the functional connectivity by considering the characteristic of brain signals that are acquired by various brain regions at the same time. PLV calculates the differences between two signal phases by extracting the components of a phase for EEG data independently. In this study, we extracted signals between -1 and 3 seconds that have an executive phase in the experimental protocol.

In this study, we analyzed the estimated matrix by PLV for each subject after preprocessing EEG data. We obtained the functional connectivity between each channel and all other channels (63) by considering all of the used channels (64) in this study (64×64–64). Next, the synchronization values between two channels were applied according to a threshold (density = 0.2783). This threshold was measured by 100 random graphs, and the details were suggested in a previous study [32]. Finally, we analyzed only significant links of the estimated matrix.

Next, we applied the brain network properties using graph theory shown below (Eqs 1 and 2) to the estimated matrix of functional connectivity per time window of 1 second. To find the time-dependent characteristics of a brain network, we analyzed the changes of each brain network properties by shifting the time window to the next 250 ms.

#### Graph theory analysis

In this paper, we modeled a brain network by adapting graph theory centered on two parameters. These parameters were acquired by the Brian Connectivity Toolbox (http://www.brain-connectivity-toolbox.net/).

First, there is degree centrality, which means the most important channel within a network in graph theory and network analysis. A channel of high centrality represents that this builds a network by connecting many channels [33]. In this study, we analyzed the node degree, which is a basic parameter of centrality [34]. We referred to the node degree as degree centrality, which is commonly used in complex network analysis.

Formula 1 indicates the degree centrality, and this formula calculates the number of connection with other channels for each channel. The degree centrality shows the number of connections emanating from a single channel in a network. The important brain regions interact with other regions and play a key role in network resilience during a bout of illness. Therefore, the high degree of centrality indicates that this channel acts as a hub in a network.

$$k_{i}=\sum_{j\in N}a_{ij}$$ (1)

*k*_*i*_ *:* Degree of a node *i*

*a*_*ij*_ *:* The connection status between *i* and *j*

Second, small-worldness is useful to measure the self-organized critical dynamics [10]. Small-worldness is possible with segregated and integrated processing of the brain [34, 35] Therefore, the high properties of small-worldness indicate that this network is able to construct the powerful and effective network. In this study, we represented the local efficiency by means of small-worldness, which measures the quantity local properties of the brain network.

Formula 2 indicates the local efficiency. The local efficiency is a kind of small-worldness parameter that considers the information transfer in the subgraph of each node *i* [36]. High values of this measurement indicate the efficiency of mutual information transfer and the tendency of clusters in subgraph of node *i*. The local efficiency indicates how much this subgraph of node *i* has fault tolerance and how well this subnetwork constructs powerful group to exchange information [37]. There are often several ways to generalize a measure to weighted networks. In the case of the local efficiency, the simplest generalization is to compute the harmonic mean of the path length on the subnetwork induced by the neighborhood of the node. This simple method, however, cannot distinguish a path between two weakly connected neighbors from a path between two strongly connected neighbors, and our generalization was designed to overcome this limitation.

$$E_{loc,i}^{w}=\frac{1}{2}\sum_{j,h\in N,j\neq i}\frac{{\left(w_{ij}w_{ih}{\left[d_{jh}^{w}\left(N_{i}\right)\right]}^{-1}\right)}^{\frac{1}{3}}}{k_{i}\left(k_{i}-1\right)}$$ (2)

*w*_*ij*_: Weight matrix value between i and *j*

$E_{loc,i}^{w}$: The local efficiency of node *i*

$d_{jh}^{w}\left(N_{i}\right)$: The length of the shortest path between j and h that contains only neighbors of *i*

### Statistical analysis for temporal brain network

In this paper, we compared the statistical difference between the brain network properties of each time window and of the baseline and then normalized data of each time windows per parameter by a one-sample Kolmogorov-Smirnov test.

The Kolmogorov-Smirnov Goodness of Fit Test is a common normalization test to confirm whether data is parametric or nonparametric. This test distinguishes whether a sample distribution is suitable for an assumed probability distribution by comparing the cumulative probability distribution between a sample and a population. As a result, our data did not have the normalized distribution because the number of subjects is small. Therefore, we conducted a Wilcoxon Rank-Sum test, which is a nonparametric statistical hypothesis test that considers pair difference. Therefore, we found the significant change of each time window in comparison with the baseline by a Wilcoxon Rank-Sum test.

### Select ROIs for brain networks

**Fig 2:**
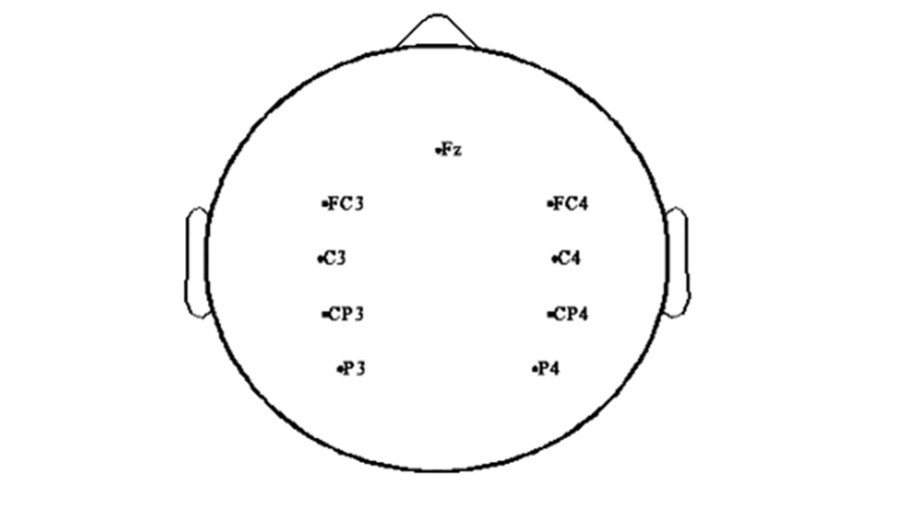
ROIs (Regions of Interest). In this study, we selected 9 ROIs in primary motor cortices (C3, C4), premotor cortices (FC3, FC4), a supplementary motor area (Fz), somatosensory cortices (CP3, CP4), and parietal areas (P3, P4) based on previous studies on motor tasks. Existing studies found meaningful characteristics on PMA, M1, SMA, the somatosensory area, and parietal area for brain networks. As in previous results, we found meaningful patterns on bilateral primary motor cortices during the active task and on bilateral somatosensory area during the MI task. For comprehension of location on ROIs, we represented 9 ROIs using the EEGlab toolbox.

We selected ROIs based on previous studies that examined motor tasks. In previous studies, neuronal signals activate in primary motor cortices, premotor cortices (PMC), the supplementary area (SMA), and parietal areas during upper extremity movement (Fig 2). Bai et al. argued that neuronal activities of the EEG spectral power were activated in the primary motor cortex and parietal area during motor executive tasks. Grefkes et al. asserted that the brain activity of stroke patients activated at the bilateral primary motor cortices, whereas that of healthy people activated at the contralateral primary motor cortex in fMRI study [2]. In addition, Grefkes et al. showed a meaningful connection among PMC, SMA, and primary motor cortices during motor execution [7]. Jin et al. identified that the local efficiency of the healthy group in the EEG study increased at the bilateral primary motor cortices and contralateral sensory area and decreased at the somatosensory cortex (SMC) in the beta band of the brain network during finger tapping [29]. There are studies in which the MI task is related to the primary motor cortex, SMA, parietal area, and cingulate cortex in the brain network for a stroke [38–42].

Based on these results, we analyzed the time-dependent brain network around the primary motor cortices, PMC, SMA, somatosensory cortex, and parietal area during two motor tasks. In accordance with the calculated value of brain network properties based on 64 channels, we interpreted a pattern of brain network properties depending on selected ROIs in the brain network of the two motor tasks. In other words, we wanted to find the meaningful regions and their roles in the brain networks according to motor tasks, although we selected ROIs.

## Results

### Significant frequency band

In this study, we normalized each brain network property of every participant on the basis of the baseline (-1 to 0s) considering the possibility of individual differences.

We found a significant frequency band to analyze temporal changes of the brain network properties during the active and MI tasks compared with the baseline. To find a significant frequency band on the brain network during motor act, the brain network properties on each time window were compared statistically with the baseline per ROIs by the Wilcoxon Rank-Sum test (p<0.05). We used this test, because our data did not have a standard normal distribution and had a small sample size (Single Sample Kolmogorov-Smirnov test, p<0.05). As a result, there were the significant changes comparing with the baseline in the high beta band at most ROIs (C3, Fz, FC4, CP3, CP4, P3, P4) at the initial motor task. The details of SMA that had the significant changes of two motor tasks in a high beta band was indicated in Table 1, and we represented significant changes of the active and MI tasks as stars in Table 1 (Wilcoxon Rank-Sum test, p<0.05). Compared to the other channels, the brain network properties on SMA in the initial motor task showed the significant difference in comparison with the baseline. Although the mu band also had significant changes in the active task, the high beta band showed statistical patterns in both the active and MI tasks. Based on these results, we analyzed the brain network properties of the two motor tasks in the high beta band.

**Table 1:** Significant Frequency Band in [-0.5–0.5s] of SMA.

	*Mu (8–12 Hz)*		*Low beta (13–20 Hz)*		*High beta (21–30 Hz)*		*Gamma (31–50 Hz)*	
	*Active*	*MI*	*Active*	*MI*	*Active*	*MI*	*Active*	*MI*
**Degree centrality**	-1.11(2.37)**	0.22(2.73)	-0.44(2.19)	0.22(1.56)	1.56(1.74)*	-1.22(0.44)**	-1.11(3.02)	-0.56(2.96)
**Local efficiency**	6.66(8.80)**	-0.26(5.29)	1.60(4.42)	1.06(3.32)	-5.98(2.86)**	2.24(1.77)**	3.53(6.85)*	-1.50(5.32)

There are the averaged value and standard deviation of each graph indices (Mean (SD)). One asterisk indicates the 5% significant level between time window in [-0.5–0.5s] and baseline (-1-0s). Two asterisks are the 1% significant level between the same times. A unit of “Local efficiency” is 10^−3^ due to the small change of this parameter between time window and the baseline.

### Characteristics of brain network properties in ROIs

In this study, we found the temporal characteristics of the brain network properties on each motor task.

#### Active task

**Fig 3:**
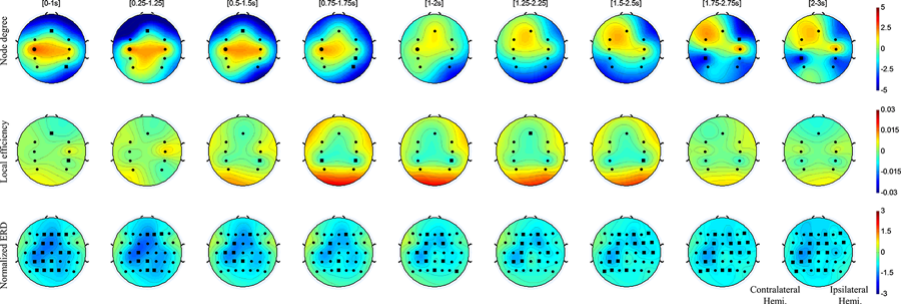
The degree centrality, local efficiency, and spectral power distribution in the active task. This figure shows the degree centrality, local efficiency, and spectral power distributions of ROIs in the active tasks. The black circles of network properties on topoplot represent significant channels that have statistical increments in comparison with the baseline (Wilcoxon Rank-Sum test, p<0.01). The black squares show significant channels that have decrement in comparison with the baseline (Wilcoxon Rank-Sum test, p<0.01). As control patterns, we represented spectral power distributions in the last row. The degree centrality and ERD patterns were significant changed in the contralateral motor cortex in comparison with the baseline (-1-0s) at the initial motor execution phase. The local efficiency decreased in bilateral somatosensory areas after the intermediate stage of the motor execution phase.

There are temporal changes of the degree centrality and local efficiency in the active task (Fig 3).

First, there were temporal changes of the degree centrality in the active task. In the initial motor execution phase, the degree centrality on bilateral primary motor cortices increased ([0-1s]-[0.5–1.5s]). In particular, the degree centrality increased significantly in the contralateral primary motor cortex ([0-1s]-[1-2s]). These results were in line with the spectral power analysis on bilateral primary motor cortices within the same time window. Also, the degree centrality in the SMA significantly decreased in the similar time window ([0-1s]-[0.5–1.5s]). Next, there were characteristics at the end of motor execution. Unlike the initial motor execution, the degree centrality in the SMA and contralateral PMC increased after the end of the motor execution phase but not significantly ([1-2s]-[2-3s]). Additionally, the contralateral somatosensory area showed significantly decreased patterns ([1.75–2.75s]-[2-3s]).

Second, there were temporal characteristics of small-worldness on the active task. The local efficiency on the ipsilateral primary motor cortex increased in the initial motor execution phase, but not significantly ([0-1s]-[0.5–1.5s]). Next, the local efficiency in the bilateral somatosensory area decreased after the intermediate step of the motor execution phase, especially in the ipsilateral area ([0.5–1.5s]-[1.5–2.5s]). The local efficiency in the SMA also decreased, but it is not statistically significant ([0.75–1.75s]-[1.5–2.5s]).

#### MI task

**Fig 4:**
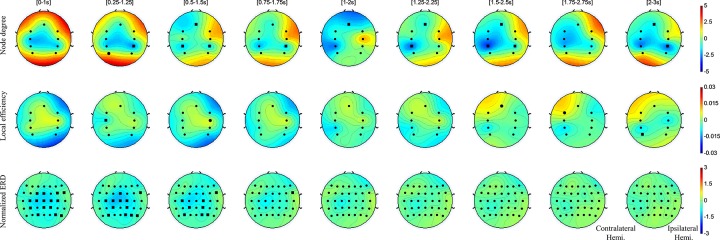
The degree centrality, local efficiency, and spectral power distribution in the MI task. This figure shows the degree centrality, local efficiency, and spectral power distributions on ROIs in MI tasks. The black circles of network properties on the topoplot represent significant channels that have a statistical increment in comparison with the baseline (Wilcoxon Rank-Sum test, p<0.01). The black squares indicate significant channels that had decrement in comparison with the baseline (Wilcoxon Rank-Sum test, p<0.01). As control patterns, we represent spectral power distributions in the last row. Degree centrality decreased in bilateral somatosensory areas after the initial motor imagery phase. The local efficiency increased the ipsilateral motor cortex just as with patterns of the active task.

There are time-dependent characteristics of the degree centrality and local efficiency in the MI task (Fig 4).

First, the degree centrality in the MI task indicated temporal patterns. There were meaningful reduction patterns on bilateral somatosensory areas at the initial motor imagery phase ([0-1s]-[0.75–1.75s]). After that, the degree centrality of the contralateral somatosensory area significantly decreased. In the same time window, the degree centrality in the ipsilateral primary motor cortex showed growing patterns, but not significantly ([0-1s]-[0.5–1.5s]). After that, the degree centrality in the SMA significantly decreased ([0.5–1.5s]-[1.5–2.5s]).

Second, there are the characteristics of the local efficiency on the MI task. In the initial motor imagery phase, the local efficiency of the ipsilateral primary motor cortex gradually increased ([0-1s]-[0.5–1.5s]). On the other hand, the contralateral somatosensory area slightly decreased, and the ipsilateral somatosensory area increased but not significantly. Besides those, there was no significant pattern.

### Correlation between brain networks and motor function

**Fig 5:**
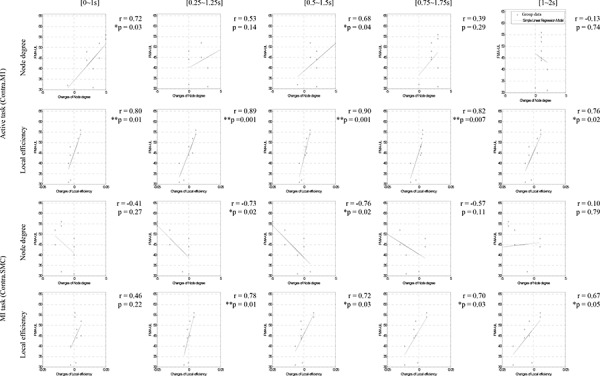
Correlations between network properties and motor functions for stroke patients (FMA-UL) during the significant motor execution period. In the active task, the degree centrality and local efficiency of the contralateral motor cortex (Contra.M1) showed positive correlation with FMA-UL at the initial motor execution phase. In the MI task, the degree centrality had negative correlations and local efficiency had positive correlations with the FMA-UL on the contralateral sensorimotor cortex (Contra.SMC) in the motor execution phase.

We analyzed the correlation between the Fugl-Meyer Assessment Scale-Upper Limb (FMA-UL) scales of the affected hand and the value of each brain network property to find the significant channel and time window related to the motor function of chronic stroke patients (Fig 5). As results, we found the significant channels between brain network property and motor function according to each motor task. The degree centrality and local efficiency of the contralateral primary motor cortex (C3) in the active task represented significant correlation with the motor function of chronic stroke patients. In the MI task, the degree centrality and local efficiency of the contralateral somatosensory area (CP3) had the significant correlation with the motor function of a stroke. The significant channels (C3 and CP3) were in the contralateral motor area of chronic patients in this study. Based on these results, we represented that the contralateral primary motor cortex and somatosensory area reflected the motor function of a chronic stroke patient in both the active and MI tasks.

## Discussion

### Significant frequency band

The active and MI tasks showed structurally significantly changes at the initial motor task phase in comparison with the baseline (-1-0s) in the high beta band (21-30Hz). To find a significant frequency band for changes of brain network properties using graph theory in two motor tasks, we performed a Wilcoxon Rank-Sum test (p<0.05) between network properties of the baseline and that of each time window. As a result, the high beta band of the sensorimotor area showed significant changes of the degree centrality and local efficiency in the initial motor task phase.

In existing studies of EEG for stroke patients, there are significant results in the beta band. Bai et al. show that there is the change of EEG spectral power of bilateral motor cortices during motor tasks in the beta band (16-24Hz) [7]. Lalo et al. argue that the beta band (13-35Hz) influences cortical sensory processing, and Gross et al. show significant differences between static and dynamic tasks in the beta band (13-24Hz) during motor executive tasks [43, 44]. As with our study, there are significant results relating to the EEG brain network in the beta band. Mima et al. argue that the beta band (14-20Hz, 22-30Hz) has a role during the movement [45]. Fallani et al. observe that the stroke have significant patterns of brain networks in the beta band (13-29Hz) [20].

In addition, there are meaningful results for motor tasks in the high beta band. Roopun et al. show the significant activities of the somatosensory and motor cortices during motor execution in the high beta frequency (20-30Hz) oscillation [46]. Zhang et al. explain that there are significant changes for coherence and Granger causality during motor executive tasks for monkeys in the high beta band (20-30Hz) [47]. Accordingly, our results in this frequency (21-30Hz) supported the findings of such existing studies that the high beta band has a specific role in network analysis during motor tasks.

### Temporal characteristics of a brain network

In this study, we found different temporal characteristics of the brain network during hand motor task of a stroke. Our main network properties are the degree centrality and the local efficiency of small-worldness. Whereas previous studies analyzed the brain network of a fixed time window, we studied the temporal characteristics of the network by shifting the time window. As a result, this study showed the different temporal patterns of the brain networks for the stroke depending on two motor tasks. These results represented that the temporal network analysis of EEG would be able to understand the characteristics of a stroke such as spectral power analysis and the different mechanism between the active task and MI task.

#### Active task

First, there were changes in the network properties of the bilateral primary motor cortices. In this study, the degree centrality and local efficiency increased on each contralateral and ipsilateral motor cortex. The increased degree centrality of the contralateral primary motor cortex are in line with previous studies. Wang et al. showed that the increased degree centrality of the contralateral primary motor cortex is correlated with the recovery of motor functions in the case of a stroke [12]. The increased degree centrality on bilateral primary motor cortices was also related to existing studies that show neural activities of bilateral primary motor cortices during motor execution of a stroke [2, 7, 13, 48]. Jin et al. found increased the local efficiency of the bilateral motor cortices for the healthy group during finger tapping [29]. Because the ipsilateral motor cortex in this study was an unaffected area, we argue that the efficiency of the ipsilateral primary motor cortex increased, as it did for the healthy group. In this study, we found that a brain network for the stroke has significant characteristics of bilateral primary motor cortices, as was found in previous studies. We hypothesized that each region constructs the subnetwork with the linked channels. The channel having high local efficiency might work in the constructed subnetwork with the linked other channels during the evoked or proposed motor task. If the subnetwork is normal, this channel might deal with the motor task by uniting the connected channels and transferring the information with other channels in subnetwork. The contralateral primary motor cortex, however, did not construct a subnetwork sufficiently because of the presence of a lesion. Therefore, the contralateral area showed a higher degree centrality and lower local efficiency than the other side because this region might just combine the weak linked channels abnormally without the information transfer. On other hand, the degree centrality and local efficiency of the ipsilateral primary motor cortex increased. We argue that these patterns of degree centrality and local efficiency indicate compensation for the affected lesion by constructing powerful subnetwork in that channel as healthy. These patterns of the ipsilateral primary motor cortex were the same in principle as those of healthy people, showing an increment of the local efficiency in the contralateral primary motor cortex.

Second, there were temporal changes of the network properties on the SMA. The degree centrality in the SMA showed a significant reduction in the initial motor execution phase. Since then, the SMA showed an increase of the degree centrality and a decrease of the local efficiency, but no statistically significant changes. The SMA had an important role in the programming and planning of motor activities [49]. We regarded that reduction of the degree centrality in the SMA as being caused by the decreased importance of the SMA after motor execution. We assumed that the SMA represented the deactivated patterns after motor execution by significantly increasing the role of SMA until execution because we compared changes of the network properties between each time window and the baseline [50]. Based on these assumptions, we regarded that the degree centrality significantly increased and the local efficiency decreased during initial motor execution in the SMA. We also assumed that the increased degree centrality in the SMA after movement is to reset the mechanism for the next motor task.

Third, there were patterns of the degree centrality of the contralateral PMC. The degree centrality of the contralateral PMC increased after the end of the motor execution phase. This result was related to the role of PMC, which is related with voluntary movement in response to sensory input by motor execution [51]. Therefore, we assumed that the results of PMC in this study were also caused by the sensory input mechanism from movement.

Fourth, there were temporal changes of the network properties of the somatosensory area. After the intermediate motor task, the local efficiency of the bilateral somatosensory area showed significantly decreasing patterns centered on the ipsilateral somatosensory area. After that, the degree centrality of the contralateral somatosensory area significantly decreased. These reductions of the degree centrality of somatosensory support the results of the study of Mauguiere et al. They argued that a deficiency in the somatosensory area is caused by peripheral neuropathy due to a cortical injury [52]. Like this result, the local efficiency of the bilateral somatosensory area decreased during motor tasks because stroke patients also have a cortical injury.

#### MI task

First, there were characteristics of the brain network properties in the bilateral somatosensory area. These areas are activated during motor imagery tasks with the presupplementary motor cortex, anterior cingulate cortex, premotor cortex, and dorsolateral prefrontal cortex [39]. These decreased patterns of degree centrality on somatosensory area are because of the motor pathways via the lateral cerebellum such as another motor pathway through area 6 (SMA, PMA) [53, 54]. This motor pathway through the lateral cerebellum sends signals for movement in the following order: somatosensory, cerebellum, ventral nucleus of the thalamus (VLc), and primary motor cortex. The effects in this motor pathway have a role for the proper movement of planned and voluntary actions because the cerebellum indicates the signals for movement with respect to motor direction, timing, and movement intensity to the primary motor cortex. Also, previous studies have argued that the MI task includes kinesthetic sensation and the cerebellum has an important role in sensorimotor integration and motor learning [55]. Therefore, we assumed that the significance of SMA was increased by the lateral motor pathway because of the rising an importance on the cerebellum during the MI task. We noted that the degree centrality in the SMA and somatosensory was decreased after motor imagery by having a weighted importance during the baseline. Therefore, the degree centrality in the SMA decreased during motor imagery relatively. It was because of that the weighted role on the cerebellum during the MI task was greater than that during the active task. In particular, the contralateral somatosensory area could have ineffective information transmission in their subnetwork by the lesion although this area constructed the weighted network with the linked channels. Thus, the local efficiency on the contralateral somatosensory area did not represent meaningfully decreased patterns in comparison with the baseline. If the informative transmission is easy during baseline, the local efficiency could show significantly the decreased patterns. In addition, the degree centrality and local efficiency on the ipsilateral somatosensory could slightly increase during the baseline as a compensation effect. Accordingly, the network properties on the ipsilateral somatosensory might have somewhat reduced patterns after motor imagery. Also, Liu et al. argued that the contralateral somatosensory area has a great role in the functional reorganization of motor functions by mental practice [56]. The significant results of the somatosensory area in this study support the concept that this area has a meaningful role in the brain network during the MI task.

Second, Hanakawa et al. argued that SMA is the predominant area for the MI task [42]. This area also relates to the planning of motor activities. The reduction of the degree centrality in the SMA indicates a diminished role for the planning of movement after the intermediate motor imagery phase. Like as the active task, the SMA could have a weighted importance during the baseline because the SMA transfers motor signals to the primary motor cortex until onset of the motor imagery. Thus, we regarded that the degree centrality on SMA decreased during motor imagery in comparison with the baseline. The degree centrality on SMA during the MI task, however, gradually decreased, unlike the active task, which showed the decreased degree centrality at initial motor execution and the increased degree centrality after that. This result has relevance with a characteristic of the MI task, which has no immediate response because it does not require real movement and is unfamiliar to task for stroke patients.

Third, there were changes in the network properties of the ipsilateral primary motor cortex in the initial motor imagery phase. MI is the mental rehearsal of motor execution [23, 38]. Just as with the active task, the local efficiency had significant patterns on the ipsilateral primary motor cortex. We regarded that these patterns of the ipsilateral primary motor cortex were compensation patterns by constructing powerful subnetwork, as with the active task, because this area did not have a lesion.

### Correlation between the brain network and motor function

There was a relation between the brain network properties of each motor task and the motor function of stroke patients. In the active task, the degree centrality and local efficiency on the contralateral motor cortex (C3) had the significant correlations with the motor functional score during the initial execution period. We found that these results were in line with those of a previous study that argues the degree centrality of the primary motor cortex of the affected hemisphere is related to FMA-UL in brain networks for strokes [21]. In the MI task, there were the significant correlations between the degree centrality or local efficiency on the contralateral somatosensory cortex (CP3) and motor function. Liu et al. showed that brain activities on the contralateral somatosensory are enhanced and have meaningful positive correlation with motor function [56]. Also, Ward et al. argued that the increased somatosensory input on the affected hand by stimulating the somatosensory area was related to motor functions of a stroke [48], and Jang et al. showed that brain activation on the contralateral somatosensory during the MI task had an important role for hand functional reorganization after mental practice training [57]. We anticipated that the characteristics of the brain network properties on the somatosensory area during the MI task had significant correlation with the motor function of the stroke in this study. Based on these results, we confirmed that the active and MI task just did not share the same mechanism correlated with the motor function although the contralateral sensorimotor area reflected the motor function of stroke patients and these regions were located nearby.

## Conclusion

In this study, we formulated three hypotheses.

First, the active task will have strong characteristics on network properties around the primary motor cortex in comparison with the MI task. Like our hypothesis, the active task showed significant changes in the degree centrality and local efficiency of the bilateral primary motor cortices, and the MI task represented significant patterns of the network properties in the SMA, ipsilateral primary motor cortex, and bilateral somatosensory cortex. Although the two motor tasks showed the significant characteristics in similar areas, the quantity of temporal network properties in the MI task were lower than those of the active task and the significant characteristics on brain network were different according to each motor task. We regarded that these characteristics indicate the possibility of the difference in the mechanism of motor execution or motor imagery. Based on these different mechanisms of each motor task, we found the significant characteristics of the brain network. Although there were different characteristics on the damaged subnetwork of the significant region on each motor task, this problem has in common on that the subnetwork was not constructed normally.

Second, the two motor tasks will have temporal change of the brain network properties. The active task had the significant changes in the degree centrality and local efficiency around the bilateral primary motor cortices during the initial motor executive phase. The MI task, also, showed the significant characteristics of two network properties around the SMA and somatosensory cortex. After that, the significant patterns disappeared. Based on these results, we argued that brain network could have temporal characteristics, such as EEG spectral power, and could show the properties of neuroscience in each region. Network analysis could apprehend the organic construction of a brain network and the location of meaningful characteristics according to each task. Therefore, we anticipate this study of the brain network might influence the rehabilitation for the stroke by understanding damaged networks that show which areas are important and have influence over motor functions.

Third, the sensorimotor area of the affected hemisphere correlates with the motor function of stroke patients. Like our hypothesis, the contralateral primary motor cortex in the active task and the contralateral somatosensory cortex in the MI task were correlated with the motor function of patients. This result indicates that the motor function and the patterns of the contralateral sensorimotor area on the brain network for the stroke have a significant correlation, and the network characteristics of each motor task have different significant areas related with motor function.

In this study, we analyzed the temporal brain network properties of chronic stroke patients in the active and the MI tasks. These results showed the possibility to interpret the temporal construction of a brain network unlike just observing activated patterns, such as EEG spectral power or brain activities, by fMRI. This analysis is useful to understand the activated patterns and the functional characteristics on brain network. However, we analyzed the brain network according to limited ROIs based on previous studies. Despite these limitations, we obtained the following significant results in this study.

1. The possibility of analyzing time-dependent changes on network properties during motor tasks.
2. The significant characteristics of the brain network during motor task in the high beta band.
3. Important roles of the ipsilateral primary motor cortex as compensation for an affected lesion.
4. The ineffective subnetwork construction on the contralateral primary motor cortex/somatosensory area with linked other channels due to a lesion during initial motor execution in the active or the MI task.
5. Significant characteristics of bilateral primary motor cortices for the active task and patterns on the somatosensory area and SMA for the MI task in the brain network. The statistically significant correlation between the network property of the significant region on each motor task and the motor function after a stroke.

Our results indicate that the characteristics of the brain networks differ according to the motor task. These results may support to apply an adaptive rehabilitation system after a stroke by understanding the functional characteristics according to the different motor task. We anticipate that these characteristics of the brain network during a stroke might help to improve rehabilitation by focusing on areas that have significant correlation with motor function depending on the motor task.
